# Estimated Incidence and Genotypes of HIV-1 among Pregnant Women in Central Brazil

**DOI:** 10.1371/journal.pone.0079189

**Published:** 2013-11-04

**Authors:** Zelma Bernardes Costa, Mariane Martins de Araujo Stefani, Yanna Andressa Ramos de Lima, Wayner Vieira de Souza, Noemia Teixeira de Siqueira Filha, Marilia Dalva Turchi, Walter Costa Borges, Clidenor Gomes Filho, Jose Vicente Macedo Filho, Ana Lucia Minuzzi, Celina Maria Turchi Martelli

**Affiliations:** 1 Faculty of Medicine, Federal University of Goias, Goias, Brazil; 2 Institute of Tropical Medicine and Public Health / Federal University of Goias, Goias, Brazil; 3 Oswaldo Cruz Foundation, Centro de Pesquisas Aggeu Magalhaes, Pernambuco, Brazil; 4 Health State Secretariat, Goias, Brazil; 5 Associacao de Pais e Amigos dos Excepcionais de Goiania - APAE, Goias, Brazil; 6 Faculty of Medicine / Federal University of Pernambuco, Pernambuco, Brazil; Alberta Provincial Laboratory for Public Health/ University of Alberta, Canada

## Abstract

**Objective:**

To estimate the incidence of HIV-1 infection among pregnant women from central-western Brazil.

**Design:**

Observational cross-sectional study.

**Methods:**

A total of 54,139 pregnant women received antenatal HIV screening from a network of public healthcare centers in 2011. The incidence of confirmed HIV-1 infection was estimated using the Serological Testing Algorithms for Recent HIV Seroconversion (STARHS) methodology and BED-capture enzyme immunoassay (BED-CEIA). The yearly incidence was calculated, and adjusted incidence rates were estimated. For a subgroup of patients, protease and partial reverse transcriptase regions were retrotranscribed from plasma HIV-1 RNA and sequenced after performing a nested polymerase chain reaction.

**Results:**

Of the participants, 20% had a pregnancy before the age of 18 and approximately 40% were experiencing their first pregnancy. Of the 54,139 pregnant women screened, 86 had a confirmed HIV-1 diagnosis, yielding an overall prevalence of 1.59 cases per 1000 women (95% CI 1.27–1.96). A higher prevalence was detected in the older age groups, reflecting cumulative exposure to the virus over time. Among the infected pregnant women, 20% were considered recently infected according to the BED-CEIA. The estimated incidence of HIV infection was 0.61 per 1000 person-years (95% CI 0.33-0.89); the corrected incidence was 0.47 per 1000 person-years (95% CI 0.26-0.68). In a subgroup of patients, HIV-1 subtype C (16.7%) was the second most prevalent form after subtype B (66.7%); BF1 recombinants (11.1%) and one case of subtype F1 (5.5%) were also detected.

**Conclusion:**

This study highlights the potential for deriving incidence estimates from a large antenatal screening program for HIV. The rate of recent HIV-1 infection among women in their early reproductive years is a public health warning to implement preventive measures.

## Introduction

Globally, approximately 34.0 million people are living with HIV. Sub-Saharan Africa (where 1.0% of adults are estimated to be infected with HIV) is the most seriously affected region of the world, followed by the Caribbean, Eastern Europe and Central Asia [1]. Worldwide, the incidence of HIV was approximately 2.5 million in 2011, which is 20% lower than the incidence rate a decade earlier, indicating a declining trend in the number of newly infected persons. An estimated 1.7 million people with HIV/AIDS live in Latin America, and approximately 40% of these cases are registered in Brazil [2]. Prevalence and incidence rates vary between and within countries, and different regions are clearly delineated [1].

In Brazil, approximately 600,000 people were living with HIV/AIDS at the end of 2000, and since then, this number has steadily increased. Since the mid-1990s, the incidence of AIDS has increased among young women as the result of the spread of the HIV-1 epidemic among heterosexual individuals [2]. Among the registered cases in 13 to 24 year-olds, the male:female ratio decreased from 3.7:1 in 1990 to 0.9:1 in 2000 [3]. According to the epidemic types defined by UNAIDS [4], the country has a concentrated epidemic because it has a high prevalence of HIV-1 (>5%) among vulnerable subpopulations, such as female sex workers, drug users and men who have sex with men [5], and less than a 1% infection rate among pregnant women in urban areas [6,7]. This epidemiologic pattern is different from the profile of a generalized epidemic, which is characterized by a high HIV-1 prevalence in the general population, as several countries on the African continent have experienced [8].

 In Brazil, a comprehensive national HIV-1 control program was implemented in the late 1980s. The program provides free antiretroviral drug therapy (ART) as well as HIV-1 counseling, testing and prophylaxis in prenatal care. It has been considered a partially successful control strategy [9]. Universal access to ART has considerably improved the life expectancy of AIDS patients and changed the profile of the HIV-1/AIDS epidemic in Brazil [10]. Since the year 2000, the prevalence of HIV-1 infection in the Brazilian population aged 15-49 years has been estimated using data from the Parturient Sentinel Surveillance studies, which are conducted nationwide biannually using a probabilistic sampling strategy. In 2004, the overall HIV-1 prevalence was 0.61% among this age group; approximately 209 thousand women and 385 thousand men (a total of 594 thousand people) were living with the infection [7,11]. 

Molecular epidemiology studies of HIV-1 indicate that the epidemic in Brazil is complex and diverse: subtype B is predominant in the country except in the southern region, where subtype C prevails [12]. Among pregnant women, recent molecular data from central Brazil have shown an unexpectedly high prevalence of subtype C viruses, which are strongly related to those circulating in southern/southeastern Brazil [13].

Estimates of HIV-1 incidence, rather than prevalence estimates, are essential for evaluating the dynamics of the epidemic in the context of the surveillance system and for implementing timely preventive measures [14,15]. Cohort studies for assessing incidence are generally costly because of the large sample size required, the need for long-term follow-up, and/or losses to follow-up [16,17]. Mathematical models using secondary prevalence data have been used to estimate newly detected cases in studies such as the UNAIDS report [18]. Recently, Serological Testing Algorithms for Recent HIV Seroconversion (STARHS) were validated in several populations for surveillance and prevention purposes [15,19–23]. The commercial BED-capture enzyme immunoassay (BED-CEIA) assay has been considered a reliable measure of HIV incidence that can be used to evaluate recent infections in diverse population groups [21,24,25]. Estimates of recently infected individuals have been calculated using STARHS in the surveillance system in the USA since 2006 [26,27] and, more recently, in several European countries [28–30]. In Brazil, although there have been estimates of HIV incidence in vulnerable subpopulations using the STARHS methodology [31–33], data on recent HIV infections among pregnant women have been scarce [34].

We have previously reported that the prevalence of HIV-1 infection was approximately 0.1% among approximately 28,000 pregnant women in the capital city in central Brazil in 2004-2005 [6]. The current study aimed to estimate the incidence of HIV-1 infection among pregnant women who underwent routine screening in a large antenatal program in Goias State in central-western Brazil in 2011. This study also describes the HIV-1 subtypes circulating in a subgroup of the pregnant women in this setting.

These findings will contribute to the understanding of the dynamics of HIV-1 transmission among young women and guide HIV control programs.

## Methods

### Study population and setting

This study aimed to estimate the incidence of HIV-1 infection and to assess the HIV-1 genotypes among pregnant women who received prenatal screening at public health clinics in Goias State in central Brazil (population ~ 4.0 million) between January and September 2011 [35]. 

Detailed information about the Program for the Protection of Pregnant Women (PPPW), which was implemented in Goias State in 2004, has been published previously [6]. Briefly, PPPW is a comprehensive screening program that works in partnership with the central laboratory (IDP/APAE) and covers approximately 80% of the pregnant women who receive public health services. Screening is available to pregnant women regardless of past HIV-1 diagnoses. As part of this program, data about demographic characteristics and obstetric history are collected at enrollment, and HIV-1 screening is offered free of charge to all pregnant women on a voluntary basis (i.e., using an “opt-in” strategy). HIV-1 screening is conducted by performing ELISA tests on eluates of dried blood spots, and confirmatory tests are performed on venous blood samples [36]. All of the tests are performed in one central laboratory located in the state capital (Goiania City), where the samples are stored for quality control and retests. In the current study, women with confirmed cases of HIV-1/AIDS were tested for recent HIV-1 infection using a commercial BED-CEIA [37]. In compliance with the Brazilian Department of STD, AIDS and Viral Hepatitis, HIV-1 positive pregnant women are compulsorily registered using the official notification system. ART is offered free of charge as one aspect of vertical HIV-1 transmission control, and there is also an official registry for drug delivery [38].

### Data Collection

Demographic characteristics and obstetric data were retrieved from the electronic records of the screening program. The collected data included date of birth, ethnicity, region of residence, number of pregnancies and gestational age (in weeks). To exclude the possibility of false recent HIV-1 results, we checked all HIV-1 positive women for previous HIV-1 notification, registration in HIV-1/AIDS healthcare programs and the use of ART. The data on HIV-1/AIDS notification, CD4 count and HIV-RNA viral load were retrieved from medical records if they were available. Laboratory results within 2 months of the current screening were considered in the analyses. In addition, the participants who were recently infected according to the serological results were contacted by one of the authors (ZBC) to determine their knowledge of their HIV-1 status (date of HIV infection), their specific drug regimen and/or the medical follow-up they had received for HIV-1/AIDS.

### Laboratory procedures

#### BED-CEIA

Recent infections with HIV-1 were identified using the commercial HIV-1 BED-CEIA enzyme immunoassay test (^©^Calypte Biomedical Corporation, Portland, OR, USA) according to the manufacturer’s instructions. Briefly, eluate was retrieved from dried whole blood (obtained with a finger prick) that had been placed onto dried blood spot (DBS) collection cards and stored at the central laboratory bank (IDP/APAE, Goiania, Brazil). The elution of the samples, controls and calibrators was performed simultaneously in a 96-microwell plate using the same procedure. The positive controls and calibrators were tested in triplicate. Three 6-mm discs were punched from the specimen and control cards and placed in each microwell. Dilution buffer was added, and the elution plate was incubated for 16 hours at 4°C. Following elution, the samples, controls and calibrators were transferred to a test plate coated with goat anti-human-IgG (1:2 dilution) and incubated (60 minutes, 37°C). The plate was washed, and biotinylated HIV-1 peptide (gp41) was added (1:1001 dilution). After washing, the plate was incubated (90 minutes, 37°C) with streptavidin-peroxidase (1:1001 dilution) and TMB substrate (15 minutes) and the optical densities (OD) were recorded (450 nm wavelength, 630 nm reference wavelength). The normalized OD (ODn) values were calculated for the controls and the samples to minimize the inter-test variability. ODn was obtained by dividing the median OD of a control/sample by the median OD of the calibrator. The samples with ODn≤1.2 were retested in triplicate, and the specimens with ODn<0.8 were considered recent seroconversions.  We considered a normalized optical density value <0.8 (which corresponds to a mean duration of infection of 155 days) according to the BED-CEIA incidence test as evidence of a recent HIV-1 infection. A recent study showed that the reliability of this test was high across duplicate specimens and different laboratories[39].

#### HIV-1 genotyping

HIV-1 subtypes were identified and molecular data were obtained only for a subset of pregnant women [n=18] because of operational difficulties in recruitment. Blood EDTA samples were collected for HIV-1 *pol* gene sequencing. Genomic RNA was extracted from plasma (QIAamp® Viral RNA Mini Kit, Qiagen, Hilden, Germany), reverse transcribed into complementary DNA (cDNA) (Invitrogen) and used as the target for a nested polymerase chain reaction (nested-PCR) employing HIV-1 protease (PR) and reverse transcriptase (RT)-specific primers (K1/K2 external primers and DP10/F2 internal primers) [40]. The entire PR gene (corresponding to positions 2253-2549 relative to the HXB2 genome, GenBank accession nº K03455) and a 750-bp fragment of the RT gene (corresponding to positions 2550-3299 relative to the HXB2 genome) were amplified. After purifying the amplicons (QIAquick® PCR Purification Kit/QIAGEN, Qiagen GmbH, Hilden, Germany), genomic sequencing was performed using a BigDye Terminator (Applied Biosystems; USA; ABI Prism 3100 Genetic Analyzer, Applied Biosystems, USA). All generated sequences underwent a quality control analysis using the HIV-1 Quality Analysis Pipeline Tool (http://www.sanbi.ac.za) and a visual inspection of the alignment using Bioedit software to exclude sample mix-ups and contamination[41]. HIV-1 genetic subtypes were determined by the REGA automated genotyping tool version 2.0 (http://www.bioafrica.net/rega-genotype/html/subtypinghiv.html) and by phylogenetic inference using the neighbor-joining (NJ) method with Kimura’s two-parameter model (MEGA4 software) [42]. The HIV-1 isolates with discordant subtypes in the PR/RT fragments were analyzed with SIMPLOT 3.5.1 software (200 bp sliding window, 20 bp increments, 1000 replicates) [43]. The Genbank accession numbers of sequences obtained in this study are KC249749-KC249766 and the URFs BF1 identified in this study are: KC249749, KC249753, KC249756, KC249766. Reference sequences and their Genbank accession numbers included in the phylogenetic analysis are: Subtype B (AY173956, K03455); CRF12_BF (AF308520, AF385934); CRF17_BF (AY037281, EU581823); CRF28_BF (DQ085874, JF804809); CRF29_BF (JF804806, DQ085876); CRF38_BF (JN235962); CRF39_BF (EU735535, EU735534); CRF40_BF (EU735537, EU735539); CRF42_BF (EU170136, EU170138); CRF44_BF (AY536235, EF193891); CRF46_BF (DQ358801, AY455782); CRF47_BF (GQ372987, GU326095) and Subtype F1 (AF077336, AF005494).

### Data analyses

The demographic and obstetric data were checked for duplicates before the analyses were conducted. We used exploratory methods of data analysis to assess the distribution of numeric variables, such as age, number of pregnancies and gestational age. The prevalence of HIV-1 infection and 95% confidence intervals (CIs) were calculated for the entire group of pregnant women, taking into account age, ethnic group, number of pregnancies and gestational age at the first screening test. The chi-square test for trends or Yates’ correction was used to analyze the prevalence of HIV-1 infection in relation to the exposure variables. In addition, we performed a multivariate logistic analysis to verify the possible association between HIV-1 infection and the number of pregnancies, controlling for age.

The annual numbers of recent and pre-existing HIV-1 infections were estimated by directly extrapolating the BED-CEIA results from the 274 days of the survey period. HIV incidence and the 95% CI were calculated based on the estimated annual number of recent and pre-existing HIV-1 infections using the recommended 155-day window and an incidence formula [24,37]. First, we calculated the crude and adjusted incidence rates using the overall number of positive cases according to the BED-CEIA test (overall diagnoses). Second, we calculated the crude and adjusted incidence rates of recently infected cases according to the BED-CEIA test, excluding the women with previous HIV diagnoses or ART (newly diagnosed cases).

Briefly, the incidence calculation was performed following the recommendations of *Surveillance and Survey and the Laboratory Working Groups* (2006). The guide’s recommended adjustment factors for the sensitivity and specificity of the test were applied to estimate the incidence and conduct surveillance using the BED-CEIA. The unadjusted BED-CEIA incidence and two adjusted corrections with 95% CIs were estimated [44,45].

The prevalence for each age group was divided by the overall estimated HIV incidence to calculate the duration of infection by age using the following formula: prevalence = incidence X duration. To estimate the duration of infection, we divided the prevalence by age group by the corrected incidence (0.47 ‰). The analyses were conducted with SPSS 18.0 and Microsoft Excel 2008.

### Ethical considerations

The study protocol was approved by the Ethical Committee of the Federal University of Goias, Brazil (CEPMHA/HC/UFG; number 174/08). All of the participants (or the legal guardians of underage participants) signed an opt-in informed consent form before receiving the screening, which was part of the routine protocol. 

## Results

**Figure 1:**
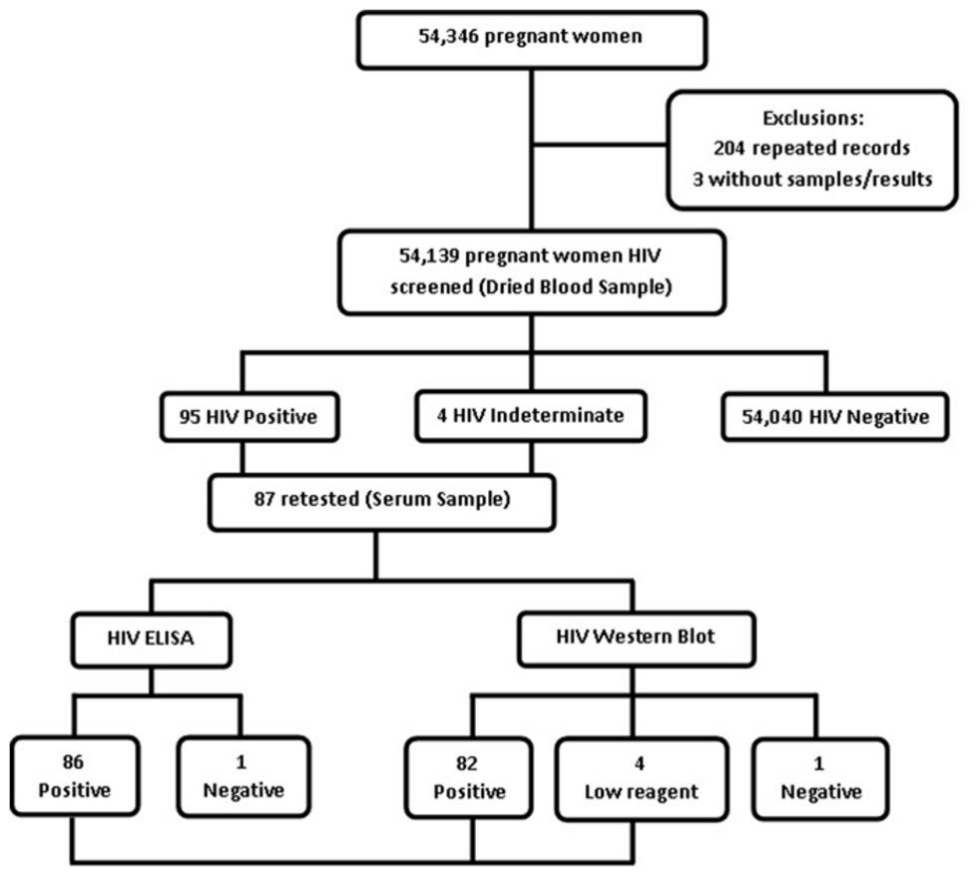
Screening strategy for HIV-1 infection among pregnant women attending antenatal clinics in central Brazil.

According to the records from the Program for the Protection of Pregnant Women, 54,346 pregnant women received HIV-1 serological screening during the study period (274 days). We excluded 204 (0.37%) records that were duplicates and three records that were missing samples or test results. Therefore, 54,139 women were eligible for inclusion in the analysis. According to the first serological screening (i.e., ELISA using eluates of dried blood spots), of the 54,139 women tested, 95 were HIV-1 seropositive and four had undetermined status. Of the 87 women who returned for ELISA and western blot confirmatory tests performed on venous blood samples, 86 had confirmed cases of HIV-1 infection (Figure 1). For the incidence study employing BED-CEIA testing, 84 filter paper samples from HIV-1 positive confirmed tests were recovered from the reference laboratory.

The 54,139 pregnant women who underwent screening had a mean age of 24.2 years (standard deviation SD=6.12) and an age range from 12 to 52 years. Adolescents between the ages of 12 and 19 comprised 26.1% of the study population. According to the exploratory data analysis, women who were 42 year old or older were considered to represent extreme values in the age distribution. Most of the participants (approximately 59%) self-reported their ethnicity as biracial or black, and 30.4% of the population was white. Approximately 40% of the pregnant women were experiencing their first pregnancy, 42.6% reported two or three previous pregnancies, 12.8% reported four or more pregnancies, and 5.2% had missing obstetric information. Most of the women (64.9%) were registered for screening at the beginning of the gestational period (≤14 weeks); 28.4% were registered later, and for approximately 8%, these data were missing.

Of the 54,139 pregnant women screened, 86 had a confirmed HIV-1 diagnosis, yielding an overall prevalence of 1.59 per 1,000 women (95% CI 1.27‰–1.96‰). Seroprevalence increased with age; the prevalence was significantly higher among older women [chi-square for trend= 11.2; degrees of freedom (df) = 2, p=0.003]. The prevalence of HIV-1 was 1.69% (95% CI 1.27-2.21) among self-identified biracial/black individuals, which was not significantly different from the prevalence of 1.21% (95% CI 0.74-1.87) among white women. In the univariate analysis, a significantly higher prevalence of HIV-1 infection was observed as the number of pregnancies increased (chi-square for trend=18.0; df=2; p<0.001). When the multivariate analysis was conducted, the number of pregnancies was still significantly associated with the outcome after controlling for age (p=0.016). Compared with one pregnancy, the OR for 2-3 pregnancies was 1.69, and the OR for 4 or more pregnancies was 2.68. The prevalence of HIV-1 was significantly lower among the women who received their initial screening before 15 weeks of gestational age compared with those who received screening later in their pregnancy at a significance level of 10% (Table 1).

**Table 1:** Prevalence of HIV-1 infection in pregnant women screened in antenatal clinic in Central Brazil, 2011.

**Characteristics**		**HIV Infection**		
	**Total**	**Positive**	**Prevalence ‰ (95% CI)^1^**	**P**
**Overall**	54,139	86	1.59 (1.27 - 1.96)	
**Age, years**				<0.001^2^
12-19	14,154	10	0.71 (0.34 - 1.30)	
20-29	29,349	51	1.74 (1.29 - 2.28)	
≥ 30	10,636	25	2.35 (1.52 - 3.47)	
**Race/Ethnicity^3^**				*0.250*
White	16,486	20	1.21 (0.74 - 1.87)	
Biracial/Black	31,923	54	1.69 (1.27 - 2.21)	
**N_o_ of pregnancies^4^**				<0.001^5^
First	21,625	20	0.92 (0.56 - 1.43)	
2–3	23,071	42	1.82 (1.31 - 2.46)	
≥ 4	6,628	22	3.32 (2.08 - 5.02)	
**Gestational age, weeks^6^**				0.095^7^
≤14	35,167	47	1.34 (0.98 - 1.78)	
>15	15,375	31	2.02 (1.37 - 2.86)	

^1^ 95% CI, 95% confidence interval

^2^ Chi Square for trend=10.88, degree of freedom =2, p<0.001

^3^ Self-reported Ethnical group; Missing data = 5730

^4^ Missing data = 2815

^5^ Chi Square for trend =18.05, df=2, P <0.001

^6^ Gestational age at the time of screening; Missing data = 3597

^7^ Chi Square =2.78; p=0.095

During the study period (9 months), 84 of the 86 women who were seropositive for HIV-1, independent of their past HIV screening, were tested by BED-CEIA. Among these women, 18 (21.4%; 18/84) were considered recently infected. Of the 18 participants with positive results on the BED-CEIA, four patients were excluded because they were considered to have long-standing HIV-1 infections. Two of these participants were already receiving ART (HIV diagnoses received in 2002 and 2006); one participant had been diagnosed in 2009 but was ART naïve. One patient could not be located and was excluded. Therefore, 14 of the 18 women were considered recently HIV-1 infected pregnant women (defined as normalized optical density values <0.8, which corresponds to a mean duration of infection of 155 days).

**Figure 2:**
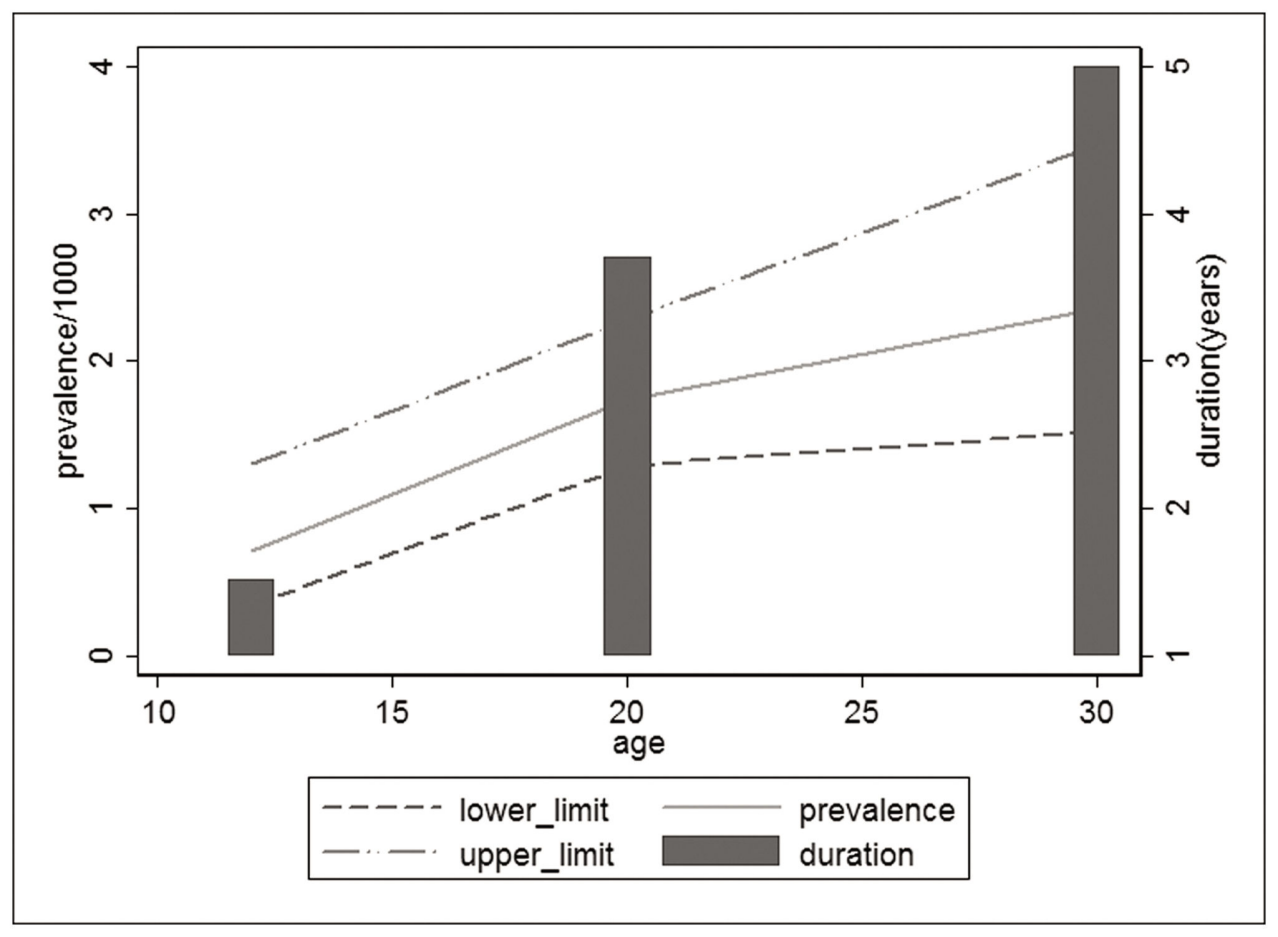
Duration of HIV infection in years among pregnant women according to age, central Brazil. Legend: The bars represent the duration in years of the infected period for each age group, and the lines represent the prevalence rates by age group and the respective 95% CIs.

First, we estimated HIV-1 incidence considering all 18 cases identified using BED-CEIA serology over the time frame of one year, which yielded 24 cases from 112 seropositive individuals. The crude incidence rate was 0.78 per 1000 person-years (95% CI 0.47‰-1.10‰). The adjusted incidence rates of HIV-1, using two different corrections, were 0.62‰ (95% CI 0.37‰-0.87‰) and 0.67 per 1000 person-years (95% CI 0.40‰-0.93‰), respectively [44,45]. The estimated incidence using the newly diagnosed cases of HIV-1 (i.e., excluding the women with previous HIV-1/AIDS diagnoses or ARV therapy) was 19 cases (17.0%) per year from 112 seropositive individuals. The crude incidence rate was 0.61 per 1000 person-years (95% CI 0.33‰-0.89‰). The adjusted incidence rates of HIV-1 using the McDougal correction [44] and the Hargrove correction [45] were 0.43 new infections per 1000 person-years (95% CI 0.24‰-0.63‰) and 0.47 per 1000 person-years (95% CI 0.26‰-0.68‰), respectively (Table 2).Figure 2 presents the prevalence rates by age group and the 95% CIs, taking into account the estimated duration of the infection. The bars represent the duration in years of the infected period for each age group, and the lines represent the prevalence rates by age group and the respective 95% CIs. The figure visualizes the speed of the changes in incidence by age. There was an increase in HIV prevalence between the early reproductive years and age 20, but the increase was less steep after age 20. The duration of HIV-1 infection was approximately 1.5 years in the youngest age group (12-19 years of age), based on the ratio of prevalence for this age strata and the adjusted incidence rate (0.71/0.47), whereas the duration of infection was 3.7 years and 5 years in older age groups.

**Table 2:** Estimation of HIV-1 incidence among pregnant women in central Brazil, 2011.

**HIV positive n=112**	**Recent infection**	**Incidence^1^ ‰/year (95% CI)**	**Incidence^2^ ‰/year (95% CI)**	**Incidence^3^ ‰/year (95% CI)**
**Overall diagnosis**	24	0.78 (0.47 - 1.10)	0.62 (0.37 - 0.87)	0.67 (0.40 - 0.93)
**Newly diagnosed**	19	0.61 (0.33 - 0.89)	0.43 (0.24 - 0.63)	0.47 (0.26 - 0.68)

Overall diagnosis - all positive HIV cases tested positive by BED-CEIA;

Newly diagnosed - number of positive HIV cases by BED- CEIA excluding women with previous HIV diagnosis / AIDS or at ARV therapy;

^1^ Crude incidence rate;

^2^ Adjusted incidence rate [44];

^3^ Adjusted incidence rate [45].

### Molecular data

HIV-1 pol was successfully genotyped in 18 of the 22 patients recruited for the molecular analysis. The samples from four patients could not be amplified by PCR. In this subsample of pregnant women who were diagnosed while receiving prenatal care, HIV-1 subtype C (16.7%, 3/18) was the second most prevalent form after subtype B (66.7%, 12/18). BF1 recombinants (11.1%, 2/18) and one case of subtype F1 (5.5%, 1/18) were also detected. Among the six cases considered recent seroconversions, 5 cases were subtype B, and one case was subtype C. 

**Figure 3:**
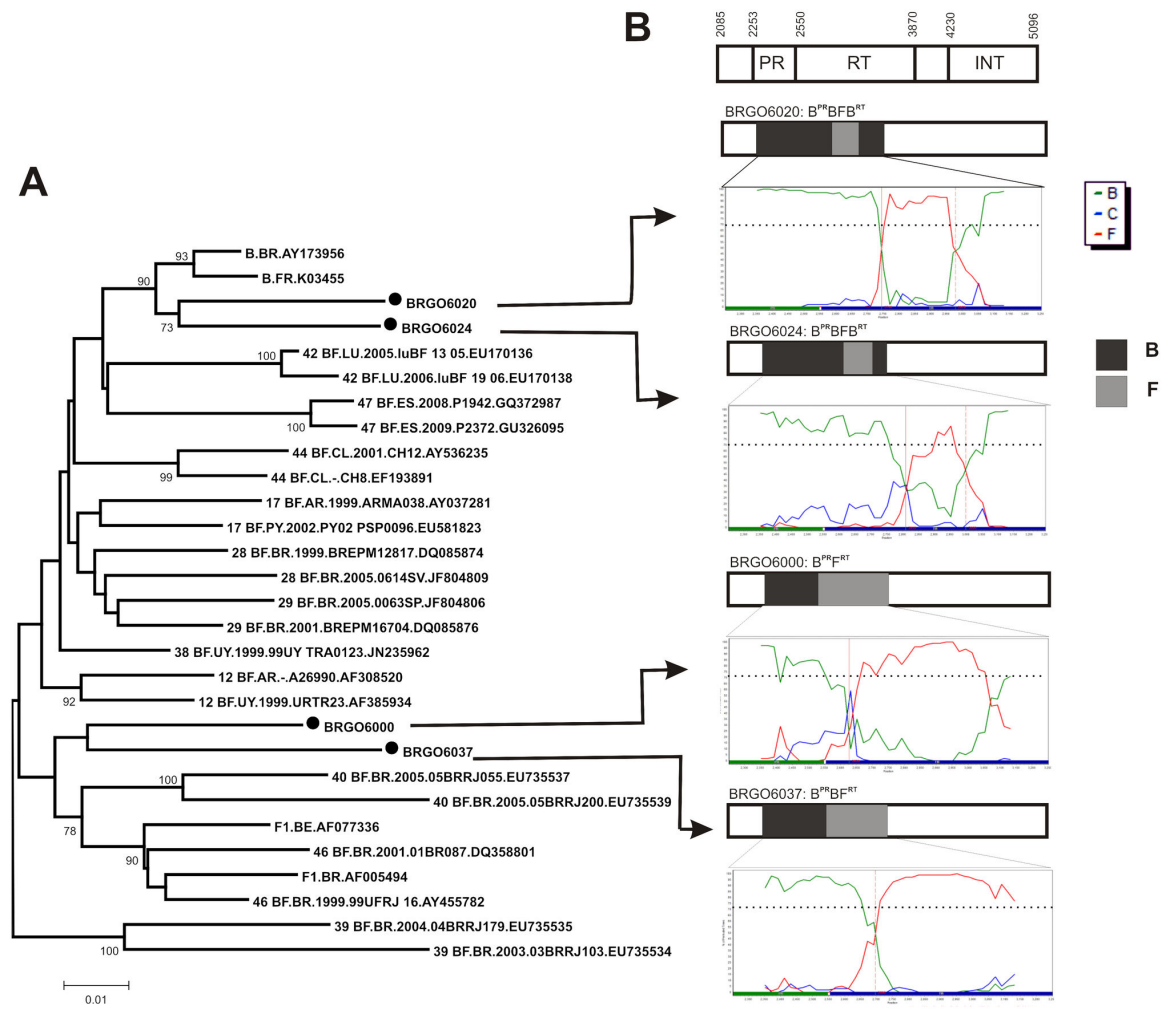
Phylogenetic and bootscanning analysis of HIV-1 unique recombinant forms (URFs) isolated from HIV-1 infected pregnant women in central Brazil. Legend: A. Phylogenetic analysis of four BF1 recombinant isolates in PR and RT genes labeled as (•). A neighbor-joining tree was constructed using reference strains from B and F1 pure subtypes and CRFs BF circulating in Brazil and in other countries, retrieved from Los Alamos database. The Genbank accession numbers of the URFs BF1 identified in this study are: KC249749, KC249753, KC249756, KC249766. Genbank accession numbers of reference sequences used in the phylogenetic analysis are: Subtype B (AY173956, K03455); CRF12_BF (AF308520, AF385934); CRF17_BF (AY037281, EU581823); CRF28_BF (DQ085874, JF804809); CRF29_BF (JF804806, DQ085876); CRF38_BF (JN235962); CRF39_BF (EU735535, EU735534); CRF40_BF (EU735537, EU735539); CRF42_BF (EU170136, EU170138); CRF44_BF (AY536235, EF193891); CRF46_BF (DQ358801, AY455782); CRF47_BF (GQ372987, GU326095) and Subtype F1 (AF077336, AF005494). B. Schematic representation of the BF1 URF mosaic structures identified in this study. Comparative bootscanning analyses were performed with known CRFs BF circulating in Brazil and none of the study sequences displayed in PR/RT a recombinant profile identical to any CRF BF already described.

The phylogenetic analysis of the four BF1 recombinant forms identified in this study was performed using reference sequences from other CRFs BF already described in Brazil (CRF28_BF, CRF29_BF, CRF39_BF, CRF40_BF and CRF46_BF) and elsewhere. The phylogenetic tree and the more detailed bootscan analyses indicated two different recombination patterns: one for BRGO6020 and BRGO6024 isolates and another for BRGO6000 and BRGO6037 isolates. In this study, all four BF1 recombinant isolates were subtype B in the PR region, and within the RT region, different BF recombination patterns were observed. Isolates BRGO6020 and BRGO6024 showed a BF1B recombination within the RT region and only a small fragment (approximately 200 bp) of subtype F1 origin. These sequences clustered with subtype B sequences. The other two BF1 recombinant isolates identified (BRGO6000 and BRGO6037) clustered with the CRF 40 BF and CRF 46 BF previously identified in Brazil, and the bootscan analyses within the RT region showed a BF1 pattern with different recombination points (Figure 3).

## Discussion

In this comprehensive antenatal care program in a central-western state in Brazil, of the 54,000 pregnant women screened, the overall prevalence of HIV-1 was 1.59 per 1000 women in 2011. Similar results for the population screened in the ANC were previously reported in the state capital [6], and our result is also comparable to the prevalence rates among women in some developed European and American countries [18]. The prevalence of HIV-1 detected in central Brazil was lower than the mean prevalence of 0.4% estimated from the Parturient Sentinel Surveillance survey, which was conducted among 16,410 pregnant women between 15 and 34 year old in Brazilian states in the early 2000s [7].

In our study, the probability of recent HIV-1 infection was 0.47 per 1,000 persons per year based on BED-CEIA serology with adjustments. Approximately 20% of the screened pregnant women who were HIV-positive were recently infected, a rate similar to the figure previously reported using the STARHS algorithm in the city of São Paulo, Brazil [34]. The national French surveillance survey of recent HIV infections (2003-2006) also reported a 23.0% rate of recent seroconversion in a low-risk population. This surveillance system was created using the network of national reference laboratories [29]. Our study design was similar to the operational strategy adopted in the French surveillance study, taking advantage of a large network of health units and stored blood samples of newly diagnosed HIV patients to further re-test the samples using the STARHS methodology. In the USA, population-level surveillance data revealed that approximately 30% of the infections were recently acquired (2006-2009). Estimates of HIV incidence among individuals older than 13 years using STARHS have been calculated from the USA surveillance system data since 2004 [15,26,27]. European countries are in the process of implementing recent HIV infection surveillance as part of their strategy to monitor transmission [28,30].

The estimated HIV-1 incidence of 0.47 new infections per 1000 person-years among the pregnant women in our study was low compared with the results of several studies conducted with high-risk subpopulations in Brazil. An HIV-1 infection incidence of 0.71/100/year was detected among cocaine users in the city of São Paulo in late 1990s using a less sensitive ELISA approach [46]. A study using the STARHS methodology found an incidence of HIV-1 infection of 0.53% among drug users in the city of Rio de Janeiro in the southeastern region of Brazil [47]. In a population receiving voluntary counseling and testing services (VCTs) in Rio de Janeiro city, the incidence rates estimated using BED-CEIA in the mid-2000s were 1.17 per 100/year [25] and 1.39 per 100/year [48]. Among the patients receiving VCTs in the city of Sao Paulo, the HIV-1 incidence rate was 0.35/100/year in 2001 [31]. In the northeastern region of Brazil, the overall incidence of HIV-1 infection was 0.44%, and when the results were stratified, the rate was 2.2 times lower among the pregnant women attending VCTs [33]. In addition, lower prevalence and incidence rates of HIV-1 are generally observed among females than among males in Brazil. Furthermore, deriving HIV-1 prevalence and incidence from VTCs generally overestimates the rates of prevalence and incidence compared with population-based surveys. HIV-1 infection in Brazil is a concentrated epidemic, not a generalized epidemic because of the high prevalence of infection among vulnerable groups [3,5,6]. Consequently, there may be bias in extrapolating the HIV-1 prevalence and incidence rates among pregnant women in our setting because these results may not mirror the dynamics of HIV-1 infection in the whole population. 

 In our study group of pregnant women, the participants were young: 26% of the pregnancies occurred in women under 20 years of age. This figure is in accordance with surveys conducted in several Brazilian capitals, which indicated that 29.6% of the participants had their first pregnancy during adolescence [49]. The prevalence of HIV-1 infection usually increases among older age groups, reflecting cumulative viral exposure over time [50]. A review from the 1995-2005 decade reported racial inequality with regard to health outcomes, with poorer health indicators among black/biracial populations in different regions throughout Brazil [6,51,52]. In the USA, HIV incidence was relatively stable between 2006-2009, but there was an increase in incidence among men who have sex with men (MSM) and black/African-American individuals [15]. 

The small number of recently infected patients identified in this study limits our ability to analyze the associations with different age groups and pregnancy. Another limitation of the current study was the operational inability to assemble data on potential risk factors such as drug use and/or sexual behavior. Because our study was based on a screening program that covers a large population of pregnant women recruited in over 240 municipalities scattered throughout Goias State in central western Brazil, it was operationally infeasible to collect data related to sensitive issues (e.g., drug use and sexual behavior) in all of the participating public health settings. A case-control study would be more suitable for investigating the risk factors among these pregnant women.

Currently, subtype C and C-containing forms are responsible for more than 51% of all worldwide HIV-1 infections [53]. In Brazil, subtype C prevails in the southern region, whereas subtype B predominates in the rest of the country [54,55]. In Brazil, subtype C was first observed in the southern region; in the central western region, it was first reported in 2000 in a pregnant woman from the southern region [56,57]. A previous regional study investigating different subpopulations reported a low rate of subtype C among infected patients [58]. However, recent molecular epidemiology studies have shown a significant increase in rates of HIV-1 subtype C among pregnant women living in small interior cities in central western Brazil [13]. 

In the current study, subtype C was the second most prevalent form of infection after subtype B; BF1 recombinants were the third most prevalent genetic variant. All three women infected with subtype C were infected locally, according to the previous history of migration. In Brazil, subtype C prevails in the southern region. Long-haul truck drivers may have played a role in the introduction of subtype C to the central region of Brazil, as previous studies have described [13].

The co-circulation of HIV-1 subtype B and subtype F1 in Brazil has likely favored inter-subtype recombination. HIV-1 BF1 recombinant viruses were first described in Brazil in the early 1990s [59]. More recently, five CRFs_BF1 (CRF28, CRF29, CRF39, CRF40 and CRF46) and several URF BF1 have been described, indicating that they represent a major feature of the Brazilian epidemic [60–62]. In central Brazil, BF1 recombinants have been the second most prevalent variant following “pure” subtype B, and our findings in this study corroborate their widespread circulation. According to phylogenetic and bootscan analyses, the four BF recombinant isolates identified do not seem to belong to any previously identified CRF BF1 described in Brazil or elsewhere because of the differences in the recombination breakpoints identified (data not shown) [60–62]. However, only full genome or near full genome sequencing can clarify whether the BF1 recombinants that circulate in central western Brazil represent another CRF BF1 or URF BF1. These findings highlight the importance of monitoring and evaluating the impact of HIV-1 diversity, especially with regard to the effects of the expansion of subtype C on the disease progression and treatment outcomes in Brazil.

Our results should be interpreted with caution because the sampling method was not random. The study population might represent women who exhibit care-seeking behavior; it is likely that certain subgroups, such as drug addicts, sex workers and homeless populations, were underrepresented. Nevertheless, the current study included approximately 80% of the estimated number of pregnant women in the state (~ 95,000 per year), and the large sample size is one of the strengths of this study. The official notification system registered 6,104 pregnant women infected with HIV in 2009 in Brazil. In the state of Goias where the study was conducted 126 pregnant infected women were notified in the same year representing 2% of the country total [63].. Data on newly infected cases is not part of the Brazilian surveillance. For Brazil as a whole, home to approximately 3.13 million pregnant women, approximately 1,470 new infections per 1000 person-years (95% CI 850 - 2,130) would be expected, assuming an incidence rate similar to that observed in our study. 

In contrast to the low HIV-1 incidence among the pregnant women in central Brazil, in countries with a generalized epidemic, the prevalence and incidence estimates derived from ANC surveys generally overestimate the rates compared with the results of population-based studies [64–66].

This study highlights the potential for estimating HIV incidence using data from a large ANC screening program to aid HIV surveillance efforts. In addition, an increase in the number of HIV-1 infections among women in their early reproductive years is a public health warning sign that should trigger preventive actions. Our findings strongly support the need for more widespread testing for HIV-1 and estimate the incidence of HIV-1 to monitor the trends in viral transmission and to evaluate the impact of preventive and educational interventions in target populations such as pregnant women.
